# A cohort study of growth differentiating factor − 15 (GDF-15) and Interleukin-6 (IL-6) as biomarkers of healthy aging in older adults living with HIV

**DOI:** 10.1186/s12877-025-06891-9

**Published:** 2025-12-23

**Authors:** Miriam Dushoff, Dawn M.E. Bowdish, Alice Zhabokritsky, Leif Erik Lovblom, Marina B. Klein, Bryan Boyachuk, Julian M. Falutz, Mona Loutfy, Graham H.R. Smith, Darrell H.S. Tan, Braeden Cowbrough, Sharon L. Walmsley

**Affiliations:** 1 Department of Medicine, McMaster University, 1200 Main Street West 4th Floor MDCL 4020, Hamilton, ON L8N 3Z5 Canada; 2 Firestone Institute for Respiratory Health, St. Joseph’s Healthcare, 50 Charlton Avenue East, Hamilton, ON L8N 4A6 Canada; 3 Division of Infectious Diseases, University Health Network, 200 Elizabeth street, ON Toronto, M5G2C4 Canada; 4 Faculty of Medicine, University of Toronto, Toronto, ON Canada; 5 Biostatistics Department, University Health Network, 700 University Avenue 2nd Floor, ON Toronto, M5G 1X6 Canada; 6 Institute of Health Policy, Management and Evaluation, University of Toronto, Toronto, ON Canada; 7 McGill University Health Center, 1001 Decarie Boulevard D02.4110, Montreal, QC H4A 3J1 Canada; 8 Women’s College Hospital Research and Innovation Institute, 14 College Street Suite 501, Toronto, ON M5G K2 Canada; 9 Maple Leaf Medical Center, 14 College Street Suite 501, Toronto, ON M5G K2 Canada; 10 Division of Infectious Diseases, St. Michael’s Hospital, 30 Bond Street 4CC Room 4-179, Toronto, ON M5B 1W8 Canada; 11 Toronto General Hospital, 585 University Ave, 13N1300, Toronto, Ontario M5G 2N2 Canada; 12 University Health Network, 585 University Avenue, 13N-1300, Toronto, ON M5G 2N2 Canada; 13 Division of Geriatrics, McGill University Health Centre Montreal General Hospital, 1650 Cedar Avenue, Montreal, Quebec H3G 1A4 Canada

**Keywords:** HIV, Biomarkers, Health, GDF-15, IL-2

## Abstract

**Background:**

The median age of Canadians living with HIV will exceed 65 years in the next decade. Compared to HIV-negative counterparts, people living with HIV exhibit elevated serum levels of inflammatory markers. In the general population, increased levels of some markers are linked to comorbidity, frailty, cognitive decline, and mortality. To date there are no HIV-specific biomarkers to measure and monitor healthy aging or to serve as endpoints in the evaluation of relevant interventions. This study aimed to evaluate inflammatory markers as indicators of healthy aging in older people living with HIV.

**Methods:**

We analyzed data from 268 consenting participants enrolled in a sub-study of the Correlates of Healthy Aging in Geriatric HIV (CHANGE HIV) study. Serum samples, demographic information and healthy aging data were collected at cohort entry. Data from one participant with HIV RNA > 200/ml were excluded. Serum levels of the biomarkers CRP (C-reactive protein), D-dimer, GDF-15 (growth differentiating factor-15), and IL-6 (interleukin-6) were quantified using ELISA and compared primarily to scores on the Rotterdam Healthy Aging Score (HAS). Statistical analyses were conducted using Spearman’s correlation and a multivariate regression model which included covariates age, race, sex, years living with HIV, body mass index and CD4 count nadir.

**Results:**

Participants had a median age of 72 years (range 65–81), and were predominantly male (92%) and White (79%). They had been living with HIV for a median of 31 years and were representative of the main CHANGE HIV study cohort. The participants had a median HAS of 12 (range 3–14). The median (IQR) for GDF-15 and IL-6 were 1810 (1310–2700) pg/ml and 3.49 (2.43–5.77) pg/ml respectively. GDF-15 and IL-6 levels were negatively associated with the HAS (*R* = -0.35 and − 0.27), *p* < 0.001 for both comparisons. The correlation persisted after adjustment for covariates. CRP (median 1.75, IQR 0.711, 4.29) ug/ml and D-Dimer (median 0.362, IQR 0.209–0.846) ug/ml did not show a clear association with HAS.

**Conclusions:**

Our results suggest further evaluation of GDF-15 and IL-6 as potential biomarkers for healthy aging in older adults living with HIV including studies testing the clinical utility of GDF-15 and IL-6 as outcomes in clinical care and in interventional studies.

**Trial registration:**

Clinical trial number not applicable.

**Supplementary Information:**

The online version contains supplementary material available at 10.1186/s12877-025-06891-9.

## Background

With the success of modern antiretroviral therapies and advances in other HIV-related management strategies, the median age of persons living with HIV in high income countries is projected to exceed 65 years in the next decade [1, 2]. This observed demographic shift introduces new challenges and highlights the need to identify factors that contribute to healthy aging and improve quality of life among people living with HIV [3, 4]. Despite life expectancy nearing that of the general population, people living with HIV face a higher burden of comorbidities, geriatric syndromes and lower quality of life [5–7]. There are no HIV-specific healthy aging indices or biomarkers currently used in clinical care or as endpoints in interventions targeting healthy aging.

Inflammatory markers are molecules upregulated in response to cell and tissue injury or infection, and identified in the overall population as indicators of health and aging [8]. In the general population, increased C-reactive protein (CRP) and interleukin 6 (IL-6) are linked to frailty, loss of physical function, decreased lung function, cardiovascular disease (CVD), cancer, and numerous other health conditions [8, 9]. D-Dimer is a sensitive marker of coagulation activation, a process exacerbated in aging [10]. GDF-15, a member of the transforming growth factor-β superfamily, was originally described as macrophage inhibitory cytokine‐1 and was shown to suppress inflammatory activation in macrophages, thus potentially linking it to innate immunosenescence [11–14]. Although its pathophysiologic role in aging is unclear, many studies have proposed an anti-inflammatory role showing it attenuates aging-mediated local and systemic inflammation in humans and mouse models [15, 16]. While age-associated rises in GDF-15 concentrations is likely an adaptive reaction to increasing stresses, it remains unclear whether chronic elevation in itself could be detrimental to health [17, 18].

Inflammatory markers (notably CRP, IL-6 and D-dimer) are elevated in persons with uncontrolled HIV replication and may not normalize despite effective antiretroviral therapy [19–21] reflecting a state of chronic inflammation. These elevated markers are associated with an increased risk on non-AIDS related comorbidity. In people living with HIV, elevated CRP and IL-6 are linked to increased CVD risk and functional impairment. Elevated plasma D-dimer levels are strong predictors of mortality and GDF-15 has been linked to an increased risk of comorbidities including CVDs and neurocognitive impairment [5, 19, 22–25].

Reliable biomarkers that correlate with healthy aging outcomes and are sensitive to change could assist in monitoring health in the clinical setting and serve as surrogate endpoints in trials [26]. Randomized controlled trials to evaluate novel interventions to improve health that use mortality or hospitalization as endpoints rely on large cohorts with extended follow up making them impractical and costly. Further, they are too coarse to pick up the multidimensional nature of healthy aging [27]. For example, two individuals who do not die or are not hospitalized during a trial’s observation period could have very different aging trajectories, one being physically active and social with good quality of life while the other having multi-morbidity and depression. There is currently no universally accepted biomarker for measuring “healthy aging” in the general population nor in people living with HIV [28]. Identifying such markers could help to advance medical care, shape healthcare policy, and improve long-term care strategies and outcomes for older adults living with HIV—ensuring that they receive comprehensive care that adequately addresses their unique health concerns [1, 29–31]. A.

To address this issue, we measured inflammatory markers in a subset of persons living with HIV enrolled in a study assessing healthy aging. We hypothesized that individuals with better health would have lower serum levels of CRP, D-Dimer, GDF-15, and IL-6.

## Methods

### Study population

CHANGE HIV is a comprehensive prospective cohort study that has enrolled 541 persons living with HIV from 7 primary and tertiary care HIV treatment centers in Canada between July 2018 and March 2024. Participants needed to be 65 years of age or older at study entry and be able to speak and comprehend English or French. Participants were recruited during regular clinic appointments. The study involved completion of 37 questionnaires around health and aging at three visits over a period of one year and repeated at 18 month intervals. Details on the study procedures has been published [32]. The main objective of the CHANGE HIV cohort is to determine the multidimensional factors associated with healthy aging in people living with HIV.

### Inflammatory marker sub-study

#### Inclusion/exclusion criteria

We planned for an enrolment of 300 persons for the inflammatory biomarker sub-study from those participating in CHANGE HIV sites in Toronto and Montreal. Of this, 268 persons have provided written informed consent.

### Measurement of inflammatory markers

Serum samples collected at the baseline visit were stored at − 80 °C and shipped to the immunology lab of Dr. Bowdish at McMaster University, Hamilton, Canada for processing. The Ella™ Bio-Techne Automated Immunoassay System was used to measure serum levels (pg/ml) of CRP, D-dimer, GDF-15 and IL-6 from participant samples [33]. Samples remained frozen until tested and then were diluted per manufacturer instructions using the provided diluents and loaded into 72-well Bio-Techne Simple cartridges (35 µL per well). Data were extracted using the Simple Plex Explorer software. There were 4 samples for D-dimer and 9 samples for CRP that assayed outside the limit of quantification. For D-dimer, the values were above the upper limit of quantification (541,890 pg/ml) and were assigned this value. For CRP, 8 samples were below the lower limit of quantification (65,600 pg/ml) and 1 sample was above the upper limit of quantification (100,000,000 pg/ml) and they were assigned these values, respectively.

### Determination of healthy aging

Healthy aging data were collected using standardized tools. The primary outcome measure was the Rotterdam Healthy Aging Score (HAS) [34]. This is a composite metric composed of seven subcategories: chronic diseases (coronary heart disease, heart failure, stroke, Parkinson’s, diabetes, chronic obstructive lung disease, cancer and chronic kidney disease); mental health using the Center for Epidemiologic Studies depression scale (CES-D) [35]; cognitive function using the Standardized Mini Mental State Examination (SMMSE) [36]; physical function using the basic and Instrumental Activities of Daily Living (ADL) scales [37, 38]; pain using a Likert scale; social support using the 5-item Duke’s index [39] and quality of life using the Questions on life satisfaction scale [34]. Each domain is scored 0, 1, or 2. The total score thus ranges from 0 to 14 where 13–14 indicates healthy aging; 11–12 intermediate aging and 0–10 unhealthy aging. In a prior pilot study of 101 older adults living with HIV attending the clinic at the University Health Network in Toronto, this score ranged from 5 to 14 with a median score of 12 [IQR (interquartile range) 10–13]. The median score did not vary by age category or sex. According to the criteria developed by the Rotterdam group, only 39% of our participants scored in the healthy range of the score [40].

In the current study, we also assessed the following secondary measures that have been evaluated in the literature as parameters relevant to health in those aging with HIV [1, 41–43]. Cognitive function was measured using the Montreal Cognitive Assessment [44] (MoCA; scored 0–30); frailty using the Fried Frailty Phenotype [45] (FFP; scored 0–5); social support using the RAND Social Support Survey Instrument [46] (scored 5–25); loneliness using the UCLA Loneliness Scale [47] (0–60); and overall self-rated health using EQ-5D-VAS [48] (scored 0-100). All scales and questionnaires were completed at the time of serum sample collection.

### Statistical analyses

Statistical analyses were performed using R (version 4.3.2). Spearman’s rank correlation assessed associations between inflammatory markers and healthy aging measures. All variables were scored on a continuous scale. To account for additional covariates that might influence this association we constructed multivariable regression models. GDF-15 and IL-6 were log-transformed to correct for residual skew. We included the age, sex, body mass index, race, years living with HIV, and CD4 nadir as factors that could impact the relationship between the inflammatory markers and the HAS [49]. 

### Ethics approval

The study was approved by the University Health Network Ethics Review Board (CAPCR 18-6311) and the REBs of the participating centers. All individuals provided written informed consent.

## Results

Of the 268 consenting participants, 264 provided samples and data. Nine participants did not complete all measures to calculate the HAS, and serum samples were not tested for inflammatory markers for 7 participants. Data from one participant was excluded because the baseline viral load was > 200 copies/ml. Thus, the final sample size of this sub-study is 247.

The demographics of the participants are reported in Table 1. Those of the sub-study are representative of the CHANGE HIV cohort as a whole. The sub-study participants were predominantly white men who have sex with men, had a median age of 72 years and had been living with HIV a median of 31 years. As reflective of an aging HIV population, the annual income was low. Half of the cohort was single. The CD4 count at study entry was a median of 561/mm3 although the CD4 count nadir was lower at a median of 245 cells/mm3.

**Table 1:** Demographic characteristics of the participants in the inflammatory marker sub-study and main CHANGE HIV study

Participant characteristics	Sub-study (*n* = 247)	CHANGE HIV (*n* = 541)
Age (median, range)	72 (65–89)	74 (65–90)
Gender (*n*)		
Male	227 (92%)	487 (89%)
Female	20 (8%)	54 (10%)
Race (*n*)		
Asian	4 (8.5%)	15 (3%)
Black	28 (11.3%)	69 (13%)
Hispanic	7 (2.8%)	11 (2%)
White	196 (79.3)	414 (75%)
Other	12 (4.8%)	36 (7%)
Body Mass Index (median, range)	25.4 (14.5–52.3)	26.3 (14.5–52.3)
Marital status (*n*, %)		
Common law	33 (14.0%)	65 (12%)
Divorced	17 (7.2%)	20 (4%)
Married	34 (14.4%)	93 (17%)
Separated	7 (3.0%)	19 (3%)
Single	111 (47.0%)	237 (44%)
Steady partner	13 (5.5%)	29 (3%)
Widowed	21 (8.9%)	48 (9%)
Missing	11	30
Annual Income $Can (*n*, %)		
Under $20,000	43 (18.6%)	102 (19%)
$20,000 - $49,999	81 (35.1%)	197 (36%)
$50,000 - $99,999	65 (28.1%)	141 (26%)
More than $100,000	42 (18.2%)	93 (18%)
Missing	16	18
Years living with HIV (median, range)	31 (3–43)	29 (2–46)
Nadir CD4 T cell count (cells/mm³; median, range)	240 (0-1354)	245 (0 -1354)
Baseline CD4 T cell count (cells/mm³; median, range)	530 (38-2201)	561 (38-2201)
Viral load (*n*,%)		
Target not detected/< Limit of quantification (> 50 copies/mL)	228 (92.1%)	512 (97.9%)
Value recorded below (< 50 copies/mL)	17 (6.9%)	11 (2.1%)
Missing	2	18

### Parameters associated with health and aging

In Table 2, we report the median and interquartile range of scores for our parameters associated with health and aging for the subset and the main CHANGE HIV participants. Overall, for the sub-set 64% would be defined as pre-frail (scores 1 or 2) and 19% as frail (scores 3–4) as defined by the Frailty Phenotype [45]. The level of cognitive function was high (median 27; IQR 25–29) as defined by the MoCA (scores 26 or higher indicating normal) [44]. There was a low degree of loneliness (median 7, IQR 2–20) ; scores above 35 suggesting moderate loneliness) [50] and a high degree of social support (Median 67; IQR 48–88 ) [46]). Overall self-rated quality of life using Eq. 5D was high (median 80; IQR 75–90) [51].

**Table 2:** Median and ranges for scores on measures associated with health and aging

Measures of health (median, range)	Sub-study (*n* = 247)	CHANGE HIV (*n* = 541)
Healthy Aging Score	12 (3–14)	12 (3–14)
Eq. 5D Visual Analogue Scale	80 (0-100)	80 (0-100)
Missing =	36	70
Fried Frailty Phenotype (*n*, %)		
0	41 (17%)	125 23%)
1	81 (33%)	184 (34%)
2	65 (26%)	134 (25%)
3	33 (13%)	72 (13%)
4	10 (4%)	17 (3%)
Missing =	17 (7%)	9 (2%)
Montreal Cognitive Assessment Score (MOCA)	27 (14–30)	26.3 (12–30)
Missing =	19	44
RAND Social Support Scale	67 (0-100)	68 (0-100)
Missing =	16	107
UCLA ( University of California, Los Angeles) Loneliness Score	7 (0–60)	7 (0–60)
Missing =	30	57

**Fig. 1:**
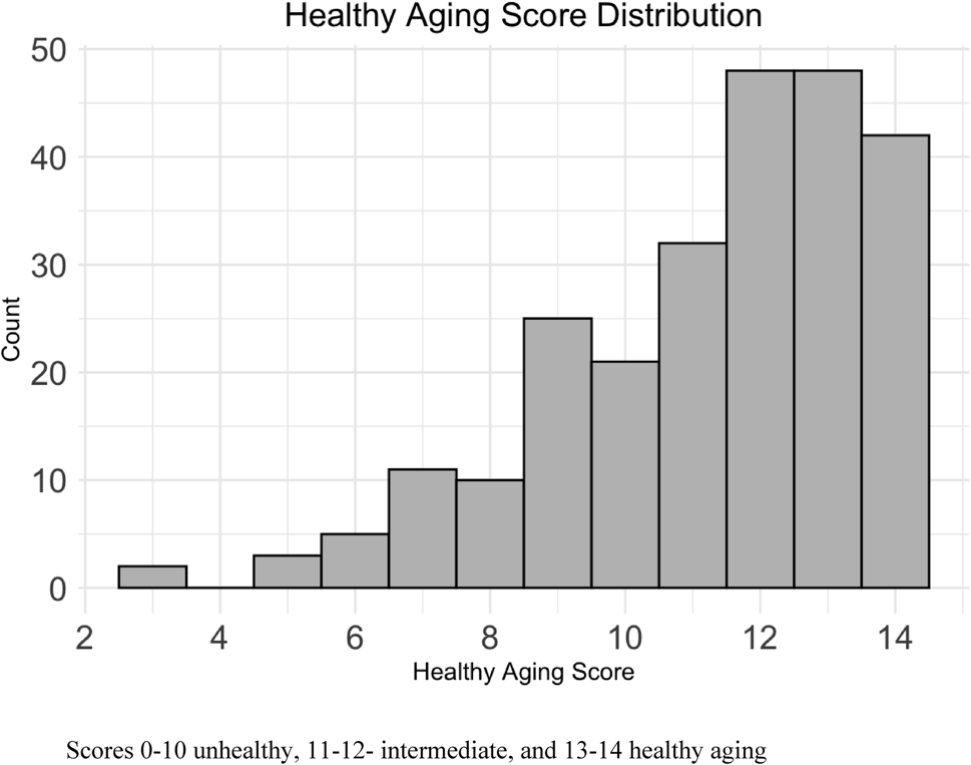
Healthy aging Score Distribution

In Fig.1 we demonstrate the distribution of scores on the HAS for our sub-study. Participants had a median HAS of 12 (range 3–14). 36.3% of participants were considered “healthy agers” (i.e., HAS > 12).

### Relationship between inflammatory mediators and healthy aging score

The median and IQR of the serum levels of the inflammatory markers- D-dimer, CRP, Il-2 and GDF-15 are described in Table 3. For each marker there was a considerable variability in the values. We investigated the univariate relationship between the inflammatory marker and HAS as shown in Table 4. CRP and D-Dimer did not show a clear association with HAS (*p* > 0.05). Elevated GDF-15 and IL-6 levels were associated with lower HAS (*p* < 0.001). In the multivariate model (Table 5) the relationship of GDF-15 and IL-6 with the HAS were maintained after adjusting for age, race, BMI, years living with HIV, and nadir CD4 cell count.

**Table 3:** Baseline serum levels of CRP, IL-6, D-dimer, and GDF-15 at entry into the cohort

Serum Biomarker Levels		
Biomarker	*n*	Serum levels (median [IQR])
CRP (µg/mL)	247	1.75 (0.721–4.39)
D-dimer (µg/mL)	219	0.389 (0.224–0.929)
GDF-15 (pg/mL)	245	1810 (1310–2700)
IL-6 (pg/mL)	226	3.49 (2.43–5.77)

*CRP* C-reactive protein, *GDF* growth differentiation factor, *IL* interleukin, *IQR* interquartile range, *n* number

**Table 4:** Assessment of the relationship between the HAS and inflammatory biomarkers (univariate). All table values were computed using spearman’s correlation

HAS vs. Biomarkers				
Biomarkers	*n*	Spearman’s rho	95% CI	*P*-value
CRP	247	-0.032	[-0.157, 0.093]	0.612
D-Dimer	219	-0.054	[-0.185, 0.079]	0.424
GDF-15	245	-0.350	**[-0.452**,** -0.237]**	< 0.001
IL-6	226	-0.273	**[-0.393**,** -0.149]**	< 0.001

*HAS* healthy aging score, *CRP* C- reactive protein, *GDF* growth differentiation factor, *IL*interleukin, *CI* confidence interval, *n* number

**Table 5:** Multivariate analysis of log-transformed GDF-15 and IL6 and HAS considering covariates, age, sex, race, BMI, years living with HIV, nadir CD4

	Outcome: log GDF15			Outcome: log IL6		
Predictors	Estimates	95% CI	*p*	Estimates	95% CI	*p*
(Intercept)	6.15	5.15–7.15	**< 0.001**	0.52	-1.17–2.21	0.55
Age, per10 year difference	0.31	0.19–0.43	**< 0.001**	0.13	-0.07–0.34	0.19
Sex [Male]	0.23	-0.01–0.47	0.06	-0.28	-0.68–0.12	0.16
Race[white]	0.12	-0.04–0.29	0.14	0.29	-0.00–0.59	0.05
BMI	-0.01	-0.03–0.00	0.07	0.03	0.01–0.05	**0.02**
Years with HIV, per 1-year difference	-0.00	-0.01–0.01	0.78	0.00	-0.01–0.02	0.75
Nadir CD4 (200–500/mm3)	-0.13	-0.26–0.00	0.05	-0.15	-0.38–0.07	0.18
Nadir CD4 (> 500/mm3)	-0.25	-0.47 – -0.03	**0.03**	-0.38	-0.75 – -0.00	**0.05**
HAS, per 1 score difference	-0.06	-0.09 – -0.04	**< 0.001**	-0.07	-0.12 – -0.03	**0.002**

*CRP* C-reactive protein, *GDF* growth differentiation factor, *IL* interleukin, *IQR* interquartile range, *n* number

**Fig. 2:**
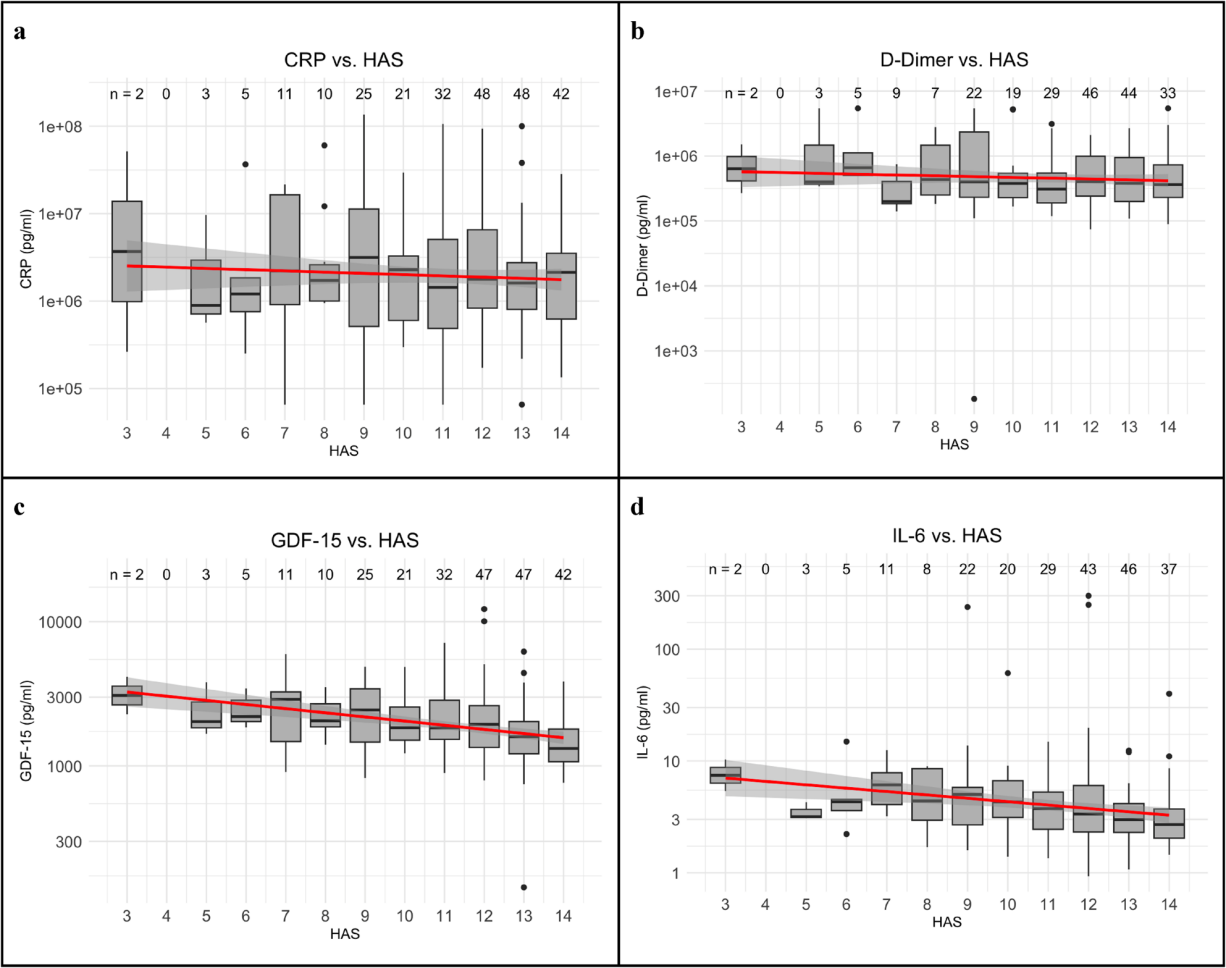
**a-d** Values of CRP (**a**), D-Dimer (**b**), GDF-15 (**c**), and IL-6 (**d**) plotted against the HAS. n values are listed above each boxplot. The median regression lines are indicated in red. Spearman’s 95% CI are as follows: CRP [-0.163, 0.087], D-Dimer [-0.185, 0.079], GDF-15 [-0.452, -0.237], IL-6 [-0.393, -0.149]. CRP- C-reactive protein, IL- interleukin, GDF- growth differentiation factor, HAS- healthy aging score, n- number, CI- confidence interval

In Fig.2 we display the correlations between the individual scores on the HAS with the levels of the inflammatory biomarkers. There is a linear correlation between the HAS and the levels of both GDF-15 and IL-6- with a stronger correlation noted for the former. In contrast, no correlations between the HAS and D-dimer or CRP were observed.

Although GDF-15 levels increase naturally with age our multivariate model found that the association between GDF-15 and HAS persists even after accounting for the effect of age: (average change of -0.06 log (pg/ml) per 1- score higher HAS, (95% CI [− 0.04, − 0.01]). Of note, age and nadir CD4 count were also associated with the log GDF15.

We then compared the relationship of the biomarkers to other secondary measures of health that could change with age. (Table 6, Supplemental Fig. 1). GDF-15 and IL-6 were positively correlated with frailty and GFD-15 with decreased cognitive function. Both markers were increased in those with lower quality of life as reported by the Eq. 5D. No marker was associated with social support or loneliness.

**Table 6:** Correlation of inflammatory biomarkers and other secondary measures of health and aging

Variable	CRP	D-Dimer	GDF-15	IL-6
EQ-5D VAS				
* n*	201	184	201	183
Spearman’s rho	0.002	-0.103	-0.135	-0.175
95% CI	[-0.136, 0.141]	[-0.242, 0.032]	[-0.28, 0.004]	[-0.302, -0.036]
* p*-value	0.976	0.163	0.056	0.018
Fried Frailty Phenotype				
* n*	230	204	229	210
Spearman’s rho	-0.032	-0.068	0.204	0.189
95% CI	[-0.161, 0.098]	[-0.198, 0.062]	[0.085, 0.316]	[0.055, 0.304]
* p*-value	0.628	0.332	0.002	0.006
Montreal Cognitive Assessment				
* n*	227	201	226	208
Spearman’s rho	-0.04	-0.08	-0.143	-0.127
95% CI	[-0.170, 0.091]	[-0.219, 0.06]	[-0.274, -0.015]	[-0.257, 0.00]
* p*-value	0.551	0.258	0.031	0.069
Rand Social Support Score				
* n*	231	205	229	212
Spearman’s rho	0.083	0.063	-0.059	-0.062
95% CI	[-0.047, 0.210]	[-0.081, 0.189]	[-0.189, 0.073]	[-0.204, 0.074]
* p*-value	0.212	0.371	0.376	0.371
UCLA Loneliness scale				
* n*	166	155	166	166
Spearman’s rho	-0.052	-0.097	0.083	0.093
95% CI	[-0.184, 0.082]	[-0.258, 0.067]	[-0.074, 0.231]	[-0.054, 0.229]
* p*-value	0.446	0.23	0.286	0.231

*VAS* visual analogue scale, *CI* confidence interval, *UCLA* University of California, Los Angeles, *CRP* C- reactive protein, *IL* interleukin, *GDF* growth differentiation factor, *n* number

## Discussion

We showed a small but positive and linear correlation between two markers of inflammation GDF-15 and Il-6 with an established measure of healthy aging (HAS) in a cohort of persons with HIV over 65 years of age. These markers also correlated with frailty and decreased cognitive function. We did not observe a correlation with CRP or D-dimer with these measures.

As life expectancy for people living with HIV continues to approach that of the general population [2, 52] attention is turning to the 4th pillar of the cascade of care: improving or maintaining quality of life [53]. While increasing research is focusing on interventions to improve healthy aging [54], to evaluate said interventions, we need to be able to measure “health”. There are little data on how to measure healthy aging in the context of HIV and no HIV-specific index of health. Older adults may present with more advanced HIV disease and have less immune recovery than younger adults [55], but for persons living with HIV on effective antiretroviral therapy, neither absolute CD4 cell counts, CD4/CD8 ratios nor HIV viral load are useful surrogate markers of healthy aging. There are no validated prognostic biomarkers for health although some are associated with survival [56–58].

Health has been measured using numerical counts of comorbidity [59, 60], but there are many components to healthy aging beyond the simple presence or absence of co-morbid disease and not all conditions have the same negative impact. Others have used the Frailty Phenotype [45], but this measure is limited, with a heavy focus on physical function. Guaraldi et al. developed a frailty index for use in HIV [61] to increase the multimodality of the measure which predicted survival and multimorbidity. There are numerous studies on chronic inflammation and immune activation (“inflamm-aging”) and HIV [62–65]. Although antiretroviral therapy effectively suppresses HIV replication, it does not completely eliminate the HIV-related inflammation [22, 56, 66], which may underlie the burden of comorbidity and contribute to accentuated or accelerated aging [2, 67].

In response to the WHO challenge for standardization of measures of health and aging [68] we selected The Rotterdam Healthy Aging Score; HAS [40] to examine the concept of healthy aging in the CHANGE HIV cohort [34]. The HAS was constructed using factor analysis from a prospective population-based study of 3500 Dutch participants *≥* 55 years old. In this population, Jaspers et al. reported a range of scores across the entire score continuum; a mean decrease in score with age; gender differences in domain sub-scores and correlations between scores of certain domains with each other [34]. The score was validated with clinical outcomes of hospitalization and mortality. In the main CHANGE HIV cohort and this sub-set we similarly demonstrate a range of outcomes. Although this could define health along a spectrum, the time required to complete the many components of the score makes it impractical to incorporate into regular clinical care.

Methods to evaluate health [69] could be complemented by reliable biomarkers, especially if they were responsive to change. Validated markers could be incorporated into clinical care to follow individuals over time or in research as endpoints for interventional studies [26]. There are minimal data on the association of biomarkers with healthy aging [9, 20] although some are elevated in HIV relative to the general population [21, 70], those with comorbidity [71, 72] or those with increased age.

GDF − 15 is a stress-response cytokine that is elevated in response to inflammation, oxidative stress and aging [11, 17, 73]. The observation that chronic inflammation may underlie aging and comorbidity in HIV inspired our investigation into the role of this marker in our study of healthy aging in HIV.

Wang et al. [25] found that GDF-15 levels were significantly elevated in people living with HIV (*n* = 64) with suppressed viral replication compared to healthy controls (*n* = 27) [25]. They also found that GDF-15 levels were positively correlated with age and markers of metabolic dysregulation. Our results complement these, suggesting GDF-15 as a potential biomarker for healthy aging in older adults living with HIV. In our multivariate analysis, the observed association of GDF-15 with health persisted after adjusting for age.

Elevated GDF-15 levels in the general population have been associated with numerous neurodegenerative conditions including cognitive decline and dementia including Alzheimer’s disease [12, 17, 74]. GDF-15 has also been linked to mitochondrial dysfunction and cellular stress responses in people living with HIV—both of which are implicated in neurodegenerative processes [25]. The association observed between GDF-15 and the MoCA in our study is complementary to results of a pilot study in persons living with HIV from the California NeuroAIDS Tissue Network [24]. Participants of this study underwent comprehensive neuropsychological exams. Post-mortem GDF-15 levels in their CSF and frontal cortex brain tissues were significantly elevated in those with neurocognitive impairment on tests within 12 months of death.

The association between GDF-15 and aging and age related diseases have led researchers to consider GDF-15 as a potential therapeutic target [17]. In animal disease models, monoclonal antibodies antagonizing GDF-15 have been tested to protect from cancer induced cachexia [75], and improve physical performance in primary mitochondrial myopathies. A human clinical trial is underway to test an anti-GDF-15 monoclonal antibody (ponsegromab) to target patients with cachexia with heart failure. It is unclear whether antagonism of GDF-15 in the context of aging and HIV would be beneficial.

IL-6 is a pro-inflammatory cytokine and a key component of the body’s inflammatory response [28]. IL-6 is linked to disease progression and adverse clinical outcomes (CVD, cancer and overall mortality) in persons living with HIV [5]. In a sub-analysis [49] of 9864 participants in the SMART, ESPRIT and SILCATT studies, higher IL-6 levels were associated with older age, nonblack race, higher BMI HIV replication, lower CD4 nadir, and comorbid conditions, multiple factors that could affect inflammation in HIV [28]. In the same cohorts, IL-6 was a stronger predictor of fatal events than of CVD and non-AIDS defining malignancy and was a stronger marker than either D-dimer or CRP. In our study of persons with controlled HIV replication, IL-6 was associated with our healthy aging score and with frailty. In our multivariable model controlling for age, race, BMI, CD4 nadir, race, and years with HIV the relationship with HAS and GDF-15 and IL-6 persisted. We did not include the one persons with HIV replication > 200 copies/ml to eliminate this as a confounder.

It is unclear whether age alone can result in increased levels of inflammatory biomarkers [10, 76, 77]. In the general population, some studies show aging to be associated with a state of chronic low-grade inflammation and raised markers. In a study of persons aged 20–90 years, serum cytokine levels (IL-6, CRP and TNF-R1) were greater in participants > 65 years but healthy older people (no comorbid disease and no medications) showed low serum levels [77].

Serum inflammatory markers are increased in persons with uncontrolled HIV infection but data are inconsistent as to the association of aging itself in HIV. While the general consensus is that serum inflammatory markers increase with both aging and HIV, many recent studies have struggled to show this association. Watanabe et al. did not find evidence of IL-6 and CRP increasing with age in individuals of 50 years or older on cART [21]. Similarly, Margolick et al. observed no significant differences in IL‐6 and CRP levels in successfully treated older men living with HIV and men living without HIV [78]. de Armas et al. also did not observe a difference between CRP and D-dimer levels between successfully treated people living with HIV and those without HIV and found that IL‐6 levels were lower in people living with HIV across all observed age groups [20]. Our data supports the findings that GDF-15 and IL-6 are increased in those individuals with poor health, and not simply with aging.

The multidimensional characteristics assessing healthy aging captured in the HAS have not always been accounted for in studies of inflammatory markers and aging. The effects of social factors are often harder to quantify or measure reliably and their contributions to health can be overlooked. We did not observe an independent association with scales evaluating loneliness and social support with our markers but in general there was minimal impairment in these scales overall. In contrast, other recent studies have shown that among people living with HIV, social isolation, decreased social support and depression are correlated with elevated levels of CRP, D-Dimer, and IL-6 [72, 79].

The strength of our study is the well-characterized cohort of older adults living with HIV and the simultaneous measures of biomarkers and health using validated scales. We used a multidimensional measure of health as our primary outcome. GDF-15 and IL-6 showed a small but linear association with health as measured by the HAS, a novel finding with potential clinical implications. The correlation was also observed with other measures of health including frailty. Our study does have important limitations. Our sample included a limited number of female participants, the imbalance reflecting the historical trajectory of the HIV epidemic in Canada which affected men considerably earlier [80]. As the proportion of women aging with HIV is expected to increase in the coming years, it will be important to understand whether these correlations are present in both sexes. Another limitation is that the HAS, while encompassing many dimensions of health, is a very coarse scale, with each subcategory scored from 0 to 2 and each subcategory ranked equally [34]. This lack of granularity reduces sensitivity to more subtle differences in health status. There may also be other confounders that could impact the relationship between the HAS and the biomarkers that we did not explore. Our sample size did not allow us to address individual comorbidities nor did we collect data on severity or extent of control of these conditions. Further, since comorbidities are included in the HAS, it was not appropriate to assess them independently. Although the number of participants with the lowest HAS scores was limited, one third of the cohort was defined as unhealthy aging. Finally, the study’s cross-sectional design limited our ability to establish causal relationships or responsiveness to change. A future step is to conduct longitudinal studies to see if GDF-15 and IL-6 can reliably predict healthy aging trajectories over the long-term. Confirming our observations in an independent aging population of persons with HIV would strengthen our findings.

## Conclusion

Identifying reliable biomarkers of healthy aging could facilitate the development of therapeutic interventions to mitigate disease progression, extend life expectancy, and improve quality of life and be used clinically to follow the course of aging. Using serum biomarkers to assess healthy aging offers a practical approach that reduces the time, effort, and resources required from clinicians and patients alike compared to comprehensive measures with questionnaires and physical maneuvers. Our findings suggest GDF-15 and IL-6 as potential clinical biomarkers for healthy aging in people living with HIV. A comprehensive approach that integrates biological, cognitive, and social determinants of health will be key to advancing research and developing effective strategies to support healthy aging in this growing population.

## Supplementary Information

Supplementary Material 1.

## Data Availability

The datasets used and analyzed during the current study are available on reasonable request to the corresponding author.

## Abbreviations

ADL: Activities of daily living
BMI: Body mass index
CART: Combination antiretroviral therapy
CES-D: Center for epidemiologic studies depression scale
CHANGE HIV: Correlates of healthy aging in geriatric HIV
CI: Confidence interval
CIHR: Canadian institutes of health research
CRP: C-reactive protein
CVD: Cardiovascular disease
EQ-5D VAS: - -EuroQol visual analogue scale
FFP: Fried frailty phenotype
GDF: Growth differentiating factor
GDF-15: Growth differentiation factor-15
HAS: Rotterdam healthy aging score
IL-6: Interleukin-6
IQR: Interquartile range
MMSE: Mini mental state examination
MoCA: Montreal cognitive assessment
UHN: University health network
