# Supplementary material for: A cohort study of growth differentiating factor − 15 (GDF-15) and Interleukin-6 (IL-6) as biomarkers of healthy aging in older adults living with HIV
Source: BMC Geriatr. 2025 Dec 23;26:108. doi: 10.1186/s12877-025-06891-9 (PMC12836993; doi:10.1186/s12877-025-06891-9)
Supplement: Supplementary file 1 — Supplementary Material 1. [file 12877_2025_6891_MOESM1_ESM.docx]

**Supplemental Figure 1**

Values of a) GDF-15 vs MOCA b) GDF-15 vs FFP, c ) IL-6 vs FFP. *n* values are listed above each boxplot. The median regression lines are indicated in red. Spearman’s 95% Confidence Interval are as follows: a) -0.143 ( -0.274, -0.015) b) .204 ( 0.085, 0.316) c) .189 ( 0.055, 0.304)

| **a**  **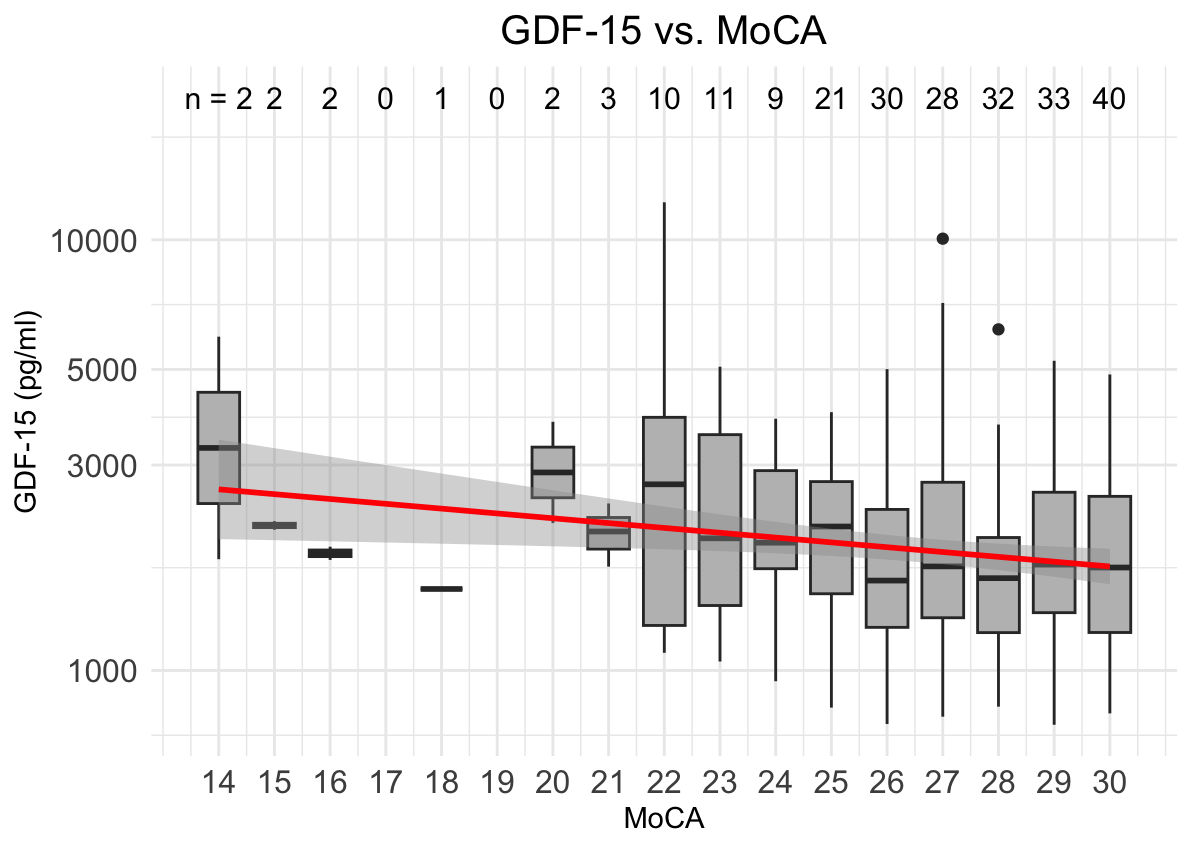** |
| --- |
| **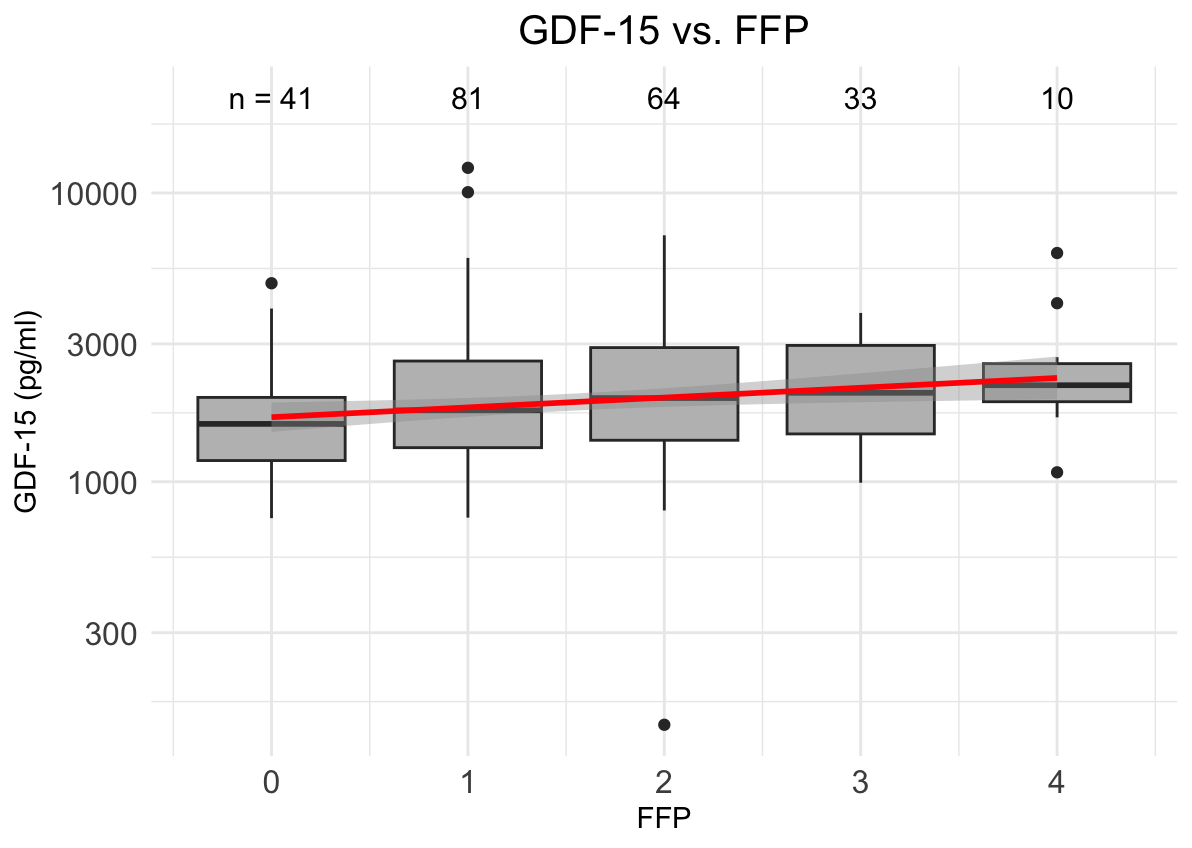** |
| **C**  **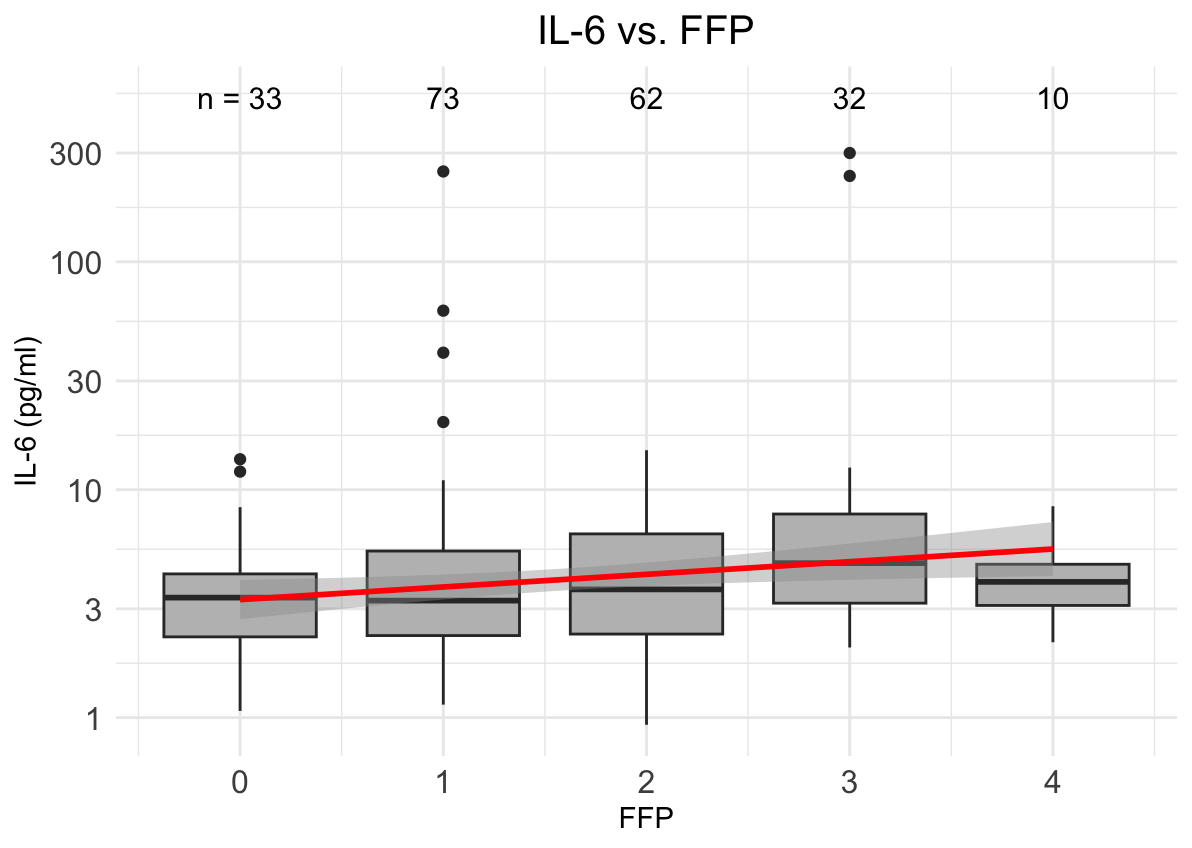** |
